# Miscellany

**Published:** 1899-07

**Authors:** 


### MISCELLANY.

Stephen T. Lee, of No. 363 South Division street, was held for the grand jury, June 13, 1899, on the charge of practising medicine illegally. The prosecution was conducted by Tracy C. Becker on behalf of the Medical Society of the County of Erie.

The attorney who appeared for Lee, asked that the defendant be discharged, stating that the prosecution had made no case. Justice

King refused to do that, whereupon Attorney Penton waived examination for his client. Lee was released on $500 bail to appear before the next grand jury.

---

GEORGE H. KINTER, the christian science healer, and James C. Saunders were held recently by the United States commissioner for the United States grand jury on a charge of manslaughter, in permitting Rolfe Saunders to die without the attendance of a regular practising physician.

Commissioner Robinson said that in justice to the defendants as well as in justice to the public at large he believed there were many facts in the case that should be presented to the higher court.

The proceedings in the case were interesting. Hon. Emory P. Close, United States district attorney, made an able argument for the prosecution and quoted many decisions bearing on the case. He was followed by Hon. Tracy C. Becker, counsel for the Medical Society of the County of Erie, who, in the course of his remarks said, it is the duty of the Medical Society of the County of Erie to see that the laws governing the practice of medicine without a license were enforced, and, he believed, the case falls within the line of the society's work.

"The only regret I have in this case," continued Mr. Becker, "is that Mrs. Kinter has been discharged. She is as guilty as the others and should have been held. There are abundant evidences that the law has been violated, even if the court should come to the conclusion that the defendants cannot be held on the charge of manslaughter; and the right of these people to apply a science to the sick should be tested with our established laws, which cover, recognise and know what science is. The fact that the Saunders boy was ill and was growing worse and that these defendants, knowing it, made no effort to learn the exact nature of the disease is sufficient to establish against them a prima facie case.

"This liberty of action and liberty of conscience talk is all rot. The highest courts in England pushed their pin through that bladder long ago and so have the highest courts in this country. It would be monstrous, yes, damnable, even, to assume that a sick person could be left to die without medical treatment and the persons in charge of him go unpunished.

"He would be an idiot—worse than an idiot—who would not take the precaution to cure a sick man by the proper method. The lungs of this dead boy were affected. That cannot be disputed. It was shown by outward symptoms. But what did these people do? They shut their eyes to all that was right and proper and called in a man who dubs himself a healer. This man is not in the business for fun. He is in it for the money, no matter what anyone else says.

"The penal code defines manslaughter as culpable negligence and those who are culpably negligent are guilty of a misdemeanor. There are laws that apply to this case and there is no question has to the defendant being guilty of manslaughter. No one can, no one ever will arise who can say that anyone who wilfully and knowingly allows a person to die because medical aid is not called has not violated the law and has not accelerated the death of that person."

---

MARIA MULLER, the so-called sympathetic healer, that treated twelve-year-old Dora Kranz, who was suffering from gangrene in the left foot, and brought her close to death's door, was tried, June 6th, before Judges Fitzgerald, Keady and Fleming in the court of special sessions in Brooklyn. She was found guilty on the technical charge of practising medicine without a license and was sentenced to five months' imprisonment in the Kings county penitentiary. To make her imprisonment sure the judges did not give her the option of paying a fine.

